# Genome‐wide meta‐analysis identifies 3 novel loci associated with stroke

**DOI:** 10.1002/ana.25369

**Published:** 2018-11-30

**Authors:** Rainer Malik, Kristiina Rannikmäe, Matthew Traylor, Marios K. Georgakis, Muralidharan Sargurupremraj, Hugh S. Markus, Jemma C. Hopewell, Stephanie Debette, Cathie L. M. Sudlow, Martin Dichgans, for the MEGASTROKE consortium and the International Stroke Genetics Consortium

**Affiliations:** ^1^ Institute for Stroke and Dementia Research, University Hospital, Ludwig Maximilian University of Munich Munich Germany; ^2^ Usher Institute of Population Health Sciences and Informatics, University of Edinburgh Edinburgh UK; ^3^ Centre for Clinical Brain Sciences University of Edinburgh Edinburgh UK; ^4^ Stroke Research Group, Division of Clinical Neurosciences University of Cambridge Cambridge UK; ^5^ INSERM U1219, Bordeaux Population Health Research Center, University of Bordeaux Bordeaux France; ^6^ Department of Neurology Institute for Neurodegenerative Disease, Bordeaux University Hospital Bordeaux France; ^7^ Clinical Trial Service Unit and Epidemiological Studies Unit, Nuffield Department of Population Health University of Oxford Oxford UK; ^8^ Munich Cluster for Systems Neurology Munich Germany; ^9^ German Center for Neurodegenerative Diseases Munich Germany

## Abstract

We conducted a European‐only and transancestral genome‐wide association meta‐analysis in 72,147 stroke patients and 823,869 controls using data from UK Biobank (UKB) and the MEGASTROKE consortium. We identified an exonic polymorphism in *NOS3* (rs1799983, p.Glu298Asp; *p* = 2.2E‐8, odds ratio [OR] = 1.05, 95% confidence interval [CI] = 1.04–1.07) and variants in an intron of *COL4A1* (rs9521634; *p* = 3.8E‐8, OR = 1.04, 95% CI = 1.03–1.06) and near *DYRK1A* (rs720470; *p* = 6.1E‐9, OR = 1.05, 95% CI = 1.03–1.07) at genome‐wide significance for stroke. Effect sizes of known stroke loci were highly correlated between UKB and MEGASTROKE. Using Mendelian randomization, we further show that genetic variation in the nitric oxide synthase–nitric oxide pathway in part affects stroke risk via variation in blood pressure. Ann Neurol 2018;84:934–939

Stroke is the leading cause of disability and the second most common cause of death worldwide. The identification of common genetic variants for vascular conditions has provided mechanistic insights, improved options for risk prediction, and facilitated the development of novel therapeutics.^1^ The MEGASTROKE consortium recently reported on the largest genome‐wide association study (GWAS) to date in >520,000 subjects from multiple ethnicities.^2^ Aside from finding novel risk loci for any stroke (AS), any ischemic stroke (AIS), and ischemic stroke subtypes, this study demonstrated marked genetic overlap with related vascular traits. However, much of the heritability of stroke remains unexplained and the biological mechanisms and pathways underlying shared genetic influences with related traits are largely elusive.

The UK Biobank (UKB) was established to improve understanding of common diseases including stroke. Participants were recruited from the general adult population and, in addition to having provided self‐reported medical history at recruitment, are followed prospectively, chiefly through linkage to their National Health Service records (http://www.ukbiobank.ac.uk).^3^ UKB recently released genotypes on >500,000 participants, thus adding to available GWAS data.

The current study aimed to identify additional susceptibility loci for stroke and obtain further insights into relevant pathways by meta‐analyzing GWAS summary statistics from UKB with data from the MEGASTROKE European‐only stratum followed by a transancestral analysis.

## Subjects and Methods

The protocol for this study received prior approval from all institutional review boards, and informed consent was obtained from each subject.

### *UK Biobank*

For definition of stroke cases, we used UKB fields 42007 and 42009, the algorithmically defined stroke outcome, including both prevalent strokes (stroke prior to recruitment) and incident strokes (first stroke diagnosed during follow‐up; http://biobank.ctsu.ox.ac.uk/crystal/docs/alg_outcome_stroke.pdf). Stroke events that were self‐reported only without corroborating evidence from medical records were excluded due to substantial uncertainty about the accuracy of stroke self‐report.^4^ Coded hospital admissions and death record data (International Classification of Diseases, 9th and 10th revision coding systems) were included based on previous work showing good accuracy of these data sources for identifying stroke cases.^5^ Participants without a stroke diagnosis were included as controls. Related participants and those of non–white‐British descent were excluded, as were single nucleotide polymorphisms (SNPs) with minor allele frequency < 0.01. The final UKB dataset consisted of 4,985 AS cases, 3,628 AIS cases, and 369,419 controls. The genetics dataset of UKB is described at https://www.biorxiv.org/content/early/2017/07/20/166298. We fitted a logistic regression model with stroke as the outcome and each SNP as a dependent variable including age, sex, principal components 1 to 10, and genotyping chip as covariates.

### *MEGASTROKE*

We used the full summary statistics from MEGASTROKE after filtering as recently described. Analyses included 67,162 AS cases, 60,341 AIS cases, 6,688 large artery stroke (LAS) cases, 9,006 cardioembolic stroke (CES) cases, and 11,710 small vessel stroke (SVS) cases.^2^

### *Genome‐Wide Association Meta‐Analyses*

We first performed a fixed‐effects meta‐analysis for AS and AIS using summary statistics from UKB and the European stratum from MEGASTROKE. The newly formed European stratum was then meta‐analyzed with the East Asian, South Asian, African American, other Asian, and Latin American strata from MEGASTROKE using a fixed‐effects model. The final dataset consisted of 72,147 AS cases (45,570 European), 63,969 AIS cases (37,845 European), and 823,869 controls (775,530 European). Single genomic control was applied for all analyses. We report on results from both the new European‐only meta‐analysis and the transancestral meta‐analysis. Genome‐wide significance was set at *p* < 5E‐8.

### *Mendelian Randomization Analysis*

To evaluate the causal association of recently published variants in the NOS3 pathway^6^ and stroke risk, we performed a 2‐sample Mendelian randomization (MR) analysis using blood pressure (BP) data from UKB as an exposure variable (systolic BP [SBP] and diastolic BP [DBP]; 318,417 subjects analyzed using a BOLT LMM model) and stroke data from the combined European‐only analysis as outcome. We used the R package “mendelianRandomization” and report on results obtained from the weighted median, inverse variance weighted, MR‐Egger, and mode‐based estimate method.

### *Expression Quantitative Trait Loci*

For lead variants at the novel loci and SNPs in linkage disequilibrium (LD; *r* ^2^ > 0.8), we queried publicly available expression quantitative trait locus (eQTL) databases GTEx7^7^ and GRASP2.^8^

## Results

We first compared effect sizes and directions of previously published lead SNPs from MEGASTROKE between UKB and the European MEGASTROKE stratum. Two low‐frequency variants at *RGS7* and *TMF4SF4* were not available in UKB, leaving 30 loci for analysis. We observed significant positive correlations in the effect sizes for both AS (*r* = 0.56, 95% confidence interval [CI] = 0.26–0.76, *p* = 0.0011) and AIS (*r* = 0.62, 95% CI = 0.34–0.80, *p* = 0.00021) between the two datasets, thus supporting our approach of meta‐analyzing them.

**Figure 1:**
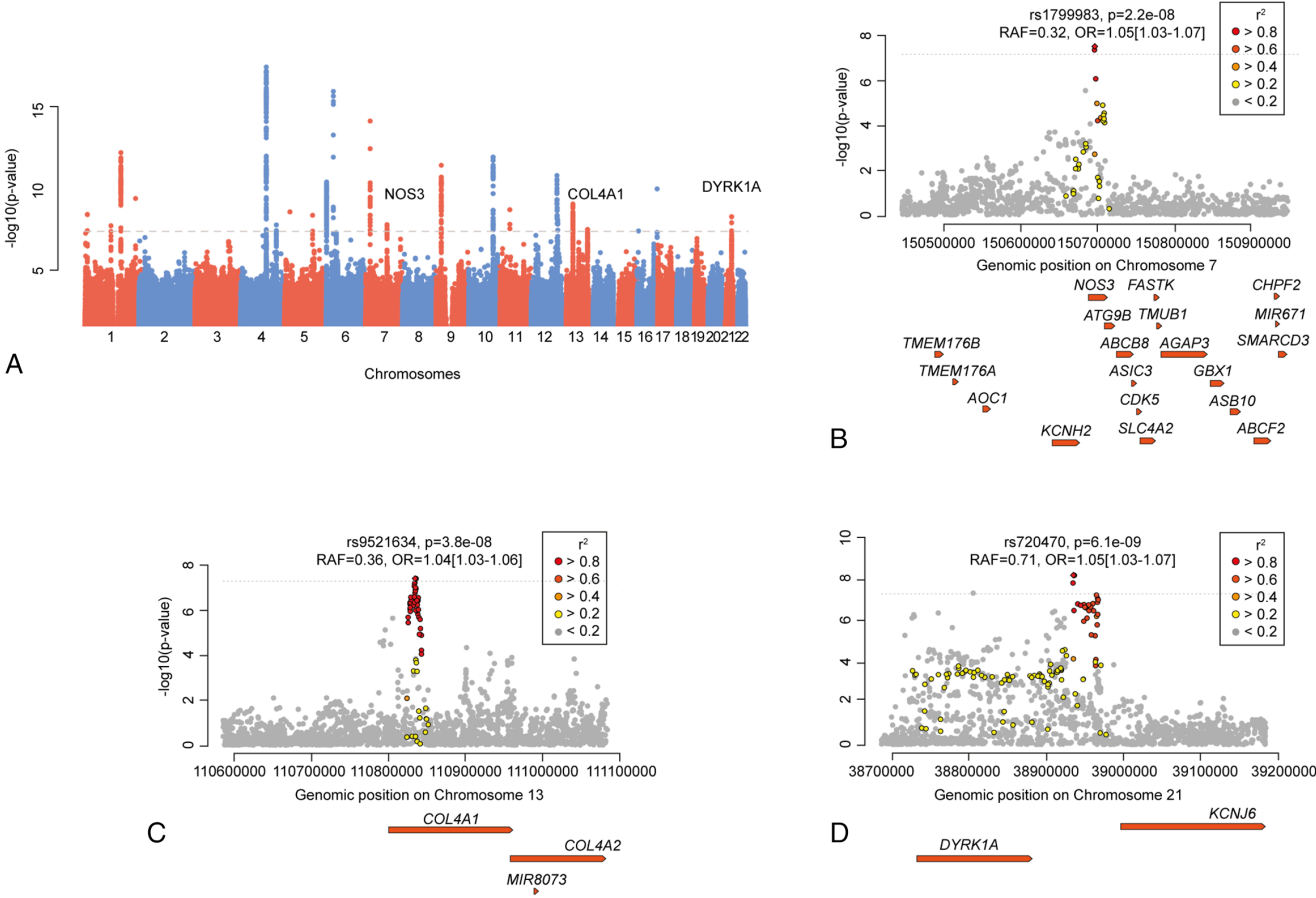
Manhattan plot and regional association plots for the 3 novel risk loci identified in the current study. (A) Manhattan plot for any stroke in the transancestral meta‐analysis. The dotted line marks the threshold of genome‐wide significance. Novel loci are marked on the top. Note that *NOS3* reached genome‐wide significance in the European‐only analysis. (B–D) Regional association plots for lead variants at the 3 novel loci: (B) *NOS3*, (C) *COL4A1*, and (D) *DYRK1A*. Shown is the region for the top signal ± 500 kb. The y‐axis represents the &minus;log(*p* value) from the European‐only fixed‐effects genome‐wide association study (GWAS) meta‐analysis (*NOS3*) or transancestral fixed‐effects GWAS meta‐analysis (*COL4A1* and *DYRK1A*). Variants in linkage disequilibrium with the lead variant are shown in red (*r* ^2^ > 0.6), orange (*r* ^2^ > 0.4), yellow (*r* ^2^ > 0.2), and gray (*r* ^2^ < 0.2). OR = odds ratio; RAF = risk allele frequency.

Fixed‐effects meta‐analysis of the UKB and MEGASTROKE datasets (up to 72,147 cases and 823,869 controls) revealed 3 novel loci reaching genome‐wide significance (*p* < 5E‐8) for association with AS in the European‐only or the full transancestral analysis (Table 1 and Fig 1A). Genomic inflation was estimated to be 1.05 for both AS and AIS (European‐only analysis). For the transethnic analyses, we found lambda values of 1.04 and 1.07, respectively. The explained phenotypic variance of the 3 novel loci is estimated to be 0.06% for AS in Europeans assuming a disease prevalence of 0.055.

**Table 1:** Results from the Fixed Effects (Transancestral and European‐Only) GWAS Meta‐Analyses.

rsID	rs1799983	rs9521634	rs720470
Gene(s)	*NOS3*	*COL4A1*	*DYRK1A*
Risk allele/reference allele	T/G	C/T	T/C
Risk allele frequency, %	32	36	71
Phenotype	AS	AS	AS
Analysis	EUR	TRANS	TRANS
MEGASTROKE OR (95% CI)	1.05 (1.03–1.07)	1.04 (1.03–1.06)	1.04 (1.02–1.06)
UKB OR (95% CI)	1.06 (1.02–1.11)	1.05 (1.01–1.09)	1.11 (1.07–1.17)
Combined OR (95% CI)	1.05 (1.04–1.07)	1.04 (1.03–1.06)	1.05 (1.03–1.07)
Combined *p* value	2.2E‐08	3.8E‐08	6.1E‐09

For each locus, the variant with the lowest *p* value in the fixed effects transancestral or European‐only meta‐analysis, respectively, is shown.

CI, confidence interval; EUR = European‐only fixed‐effects meta‐analysis; GWAS = genome‐wide association study; OR, odds ratio; TRANS = transancestral fixed‐effects meta‐analysis; UKB = UK Biobank.

**Figure 2:**
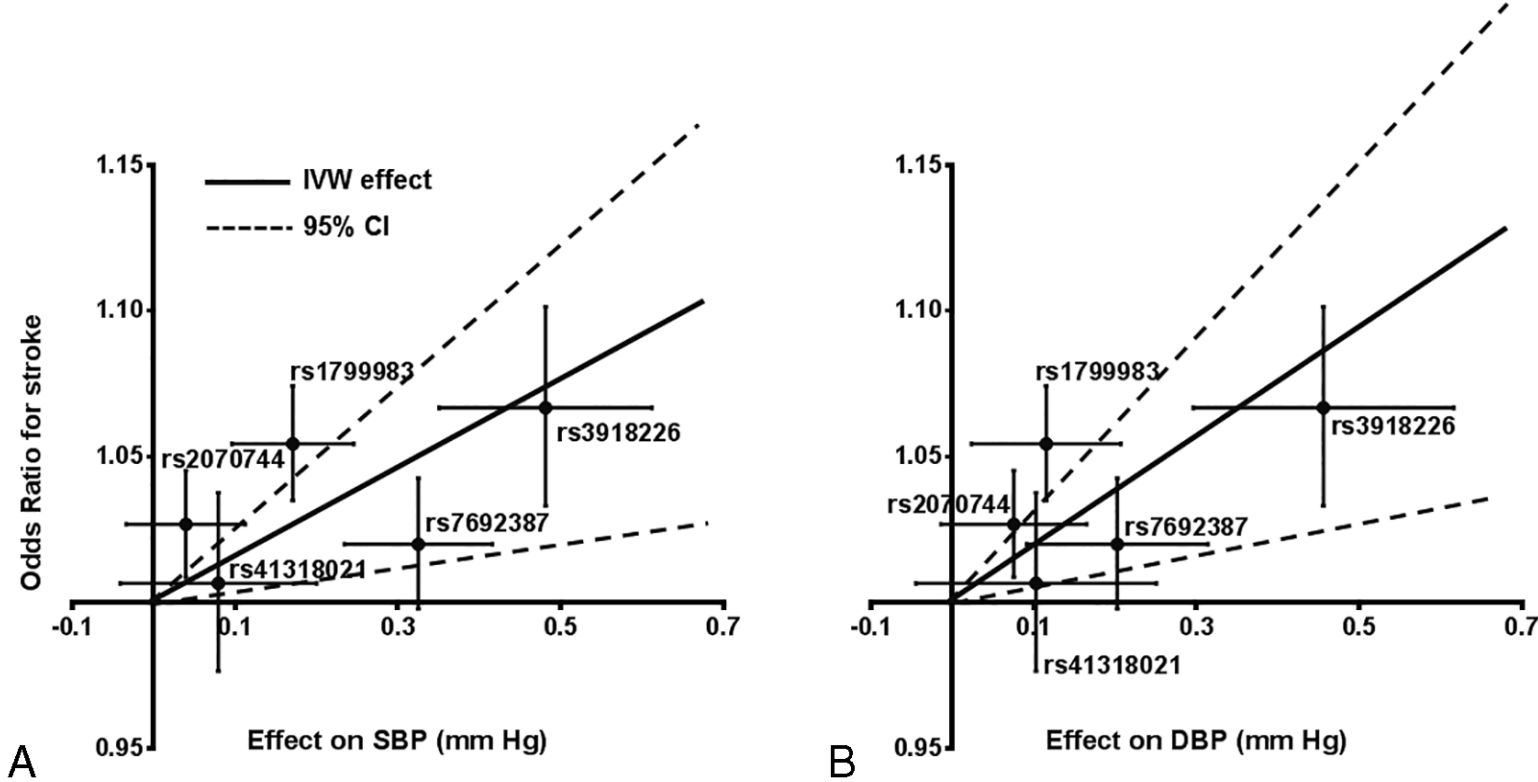
Association of common variants in the nitric oxide synthase–nitric oxide (NOS‐NO) signaling pathway with blood pressure and with any stroke. For each single nucleotide polymorphism in the NOS‐NO signaling pathway, the effect for (A) systolic blood pressure (SBP) and (B) diastolic blood pressure (DBP) is displayed on the x‐axis and the odds ratio for any stroke from the European‐only analysis is displayed on the y‐axis together with their respective standard errors. The solid and dashed lines display the inverse variance weighted (IVW) effect estimate and 95% confidence interval (CI), respectively. Estimates for SBP and DBP were derived from UKB. Estimates for stroke were derived from inverse variance fixed effects meta‐analyses of MEGASTROKE and UK Biobank.

The first finding is an exonic SNP in the nitric oxide synthase 3 (*NOS3)* gene (rs1799983, p.Glu298Asp) that was associated with AS in the European‐only analysis (*p* = 2.2E‐8, OR = 1.05, 95% CI = 1.04–1.07; see Table 1 and Fig 1B). Functional variants in *NOS3* and other genes of the NOS‐NO pathway have been associated with hypertension.^6^ Hence, we explored the possibility that the effects of genetically determined dysregulation of the nitric oxide synthase–nitric oxide (NOS‐NO) pathway on stroke risk are mediated via BP using a 2‐sample MR analysis. We selected instruments in the genes encoding proteins in the NOS‐NO pathway that exert known functional effects on NO signaling, as summarized in a recent review.^6^ The genetic instruments included variants in *NOS3* (rs1799983, rs2070744, rs3918226), *GUCY1A3* (guanylate cyclase 1, soluble alpha 3; rs7692387), and the L‐arginine transporter gene *SLC7A1* (rs41318021)^6^; rs7539120 in the nitric oxide synthase 1 adaptor protein gene *NOS1AP* was not available for analysis in UKB. Considering BP in UKB as the exposure and AS as outcome, we found significant associations using the weighted median approach (OR = 1.11, *p* < 0.0001 for SBP; OR = 1.14, *p* < 0.0001 for DBP) and inverse variance weighted (IVW) effect estimate (OR = 1.15, *p* = 0.005 for SBP; OR = 1.19, *p* = 0.002 for DBP), suggesting a causal association of the instruments with stroke via BP (Fig 2) with an estimated contribution^9^ of 4%. We found no evidence of heterogeneity in the IVW analyses, and the intercept in the MR‐Egger analyses were not significant (all *p* > 0.10), suggesting absence of significant pleiotropy. After excluding 2 potentially pleiotropic SNPs identified through PhenoScanner as being associated with coronary artery disease (rs3918226, rs7692387), the results remained significant.

We further found variants in *COL4A1* to be associated with AS (rs9521634, transancestral analysis: *p* = 3.8E‐8, OR = 1.04, 95% CI = 1.03–1.06; see Table 1 and Fig 1C); rs9521634 is situated in intron 28 but does not act as a known eQTL for any gene in available tissues, as is also true for SNPs in LD (*r* ^2^ > 0.8) with rs9521634.

The third locus resides in the intergenic region between *DYRK1A* and *KCNJ6* (rs720470, *p* = 6.1E‐9, OR = 1.05, 95% CI = 1.03–1.07; see Table 1 and Fig 1D); rs720470 serves as an eQTL for *DYRK1A* in whole blood (*p* = 9E‐4),^10^ thus identifying *DYRK1A* as a plausible causal gene at this locus.

Association results for ischemic stroke subtypes were available from MEGASTROKE. rs1799983 in *NOS3* showed the strongest association with CES (*p* = 4.6E‐5, OR = 1.09, 95% CI = 1.04–1.13; *p* > 0.1 for both SVS and LAS), whereas rs9521634 in *COL4A1* showed the strongest association with SVS (*p* = 0.0004, OR = 1.06, 95% CI = 1.03–1.10; *p* > 0.3 for CES and LAS). There was no clear association signal for ischemic stroke subtypes at *DYRK1A* (all *p* > 0.01).

## Discussion

We found rs1799983 encoding p.Glu298Asp in endothelial nitric oxide synthase (eNOS) to reach genome‐wide significance for association with AS. Nitric oxide signaling is a key regulator of vascular tone, BP, and platelet aggregation. p.Glu298Asp lowers eNOS activity by disruption of eNOS caveolar localization.^11^ Another variant, rs3918226, which is situated in the *NOS3* promoter and was found to lower promoter activity,^12^ has been shown to associate with both hypertension^12^ and coronary artery disease.^13^ rs3918226 is in low LD (*r* ^2^ = 0.17) with our lead SNP and did not reach genome‐wide significance for association with AS or AIS. However, our MR analysis suggests that the aggregate effects of common variants in the NOS‐NO pathway on stroke risk are in part mediated through blood pressure. Similar results have recently been shown for coronary artery disease using a genetic risk score comprised of 2 common variants in *NOS3* and *GUCY1A3*.^14^ Somewhat surprisingly, we found the strongest association with CES. Although this might relate to limited power in stroke subtypes, potential mechanisms underlying this association might include prothrombotic effects as well as mechanisms that are yet unknown.

Our findings further highlight a role of *COL4A1* in stroke. Collagen type IV α1 is a major constituent of the vascular basement membrane and forms heterotrimers with collagen IV α2. Rare variants in *COL4A1* cause monogenic small vessel disease, with hemorrhagic and ischemic stroke being part of the spectrum.^15^, ^16^ These mutations are associated with structural protein changes or altered expression levels of COL4A1, which interfere with the assembly, secretion, or biological function of COL4A1. Although we found no eQTLs for variants in LD with our lead SNPs in GTEX7 and GRASP2, there might be eQTLs in relevant tissues or cell types not captured by these sources. In keeping with the role of *COL4A1* in small vessel disease, we found rs9521634 to show the strongest association signal in SVS. Interestingly, common variants in the adjacent *COL4A2* gene associate with SVS,^2^, ^17^ intracerebral hemorrhage,^17^ and white matter hyperintensities.^18^ Collectively, these findings define COL4A1 and COL4A2 as key molecules in the biology of stroke and small vessel disease.

Our association results in combination with the eQTL data further point to a potential role of *DYRK1A* in stroke. *DYRK1A* encodes a dual‐specificity tyrosine‐phosphorylation–regulated kinase 1A that has recently been shown to regulate angiogenic responses in vascular endothelial cells.^19^ *Dyrk1a* heterozygous mice exhibit defects in retinal vascularization, and DYRK1A was found to positively regulate vascular endothelial growth factor–dependent transcriptional responses in endothelial cells.^19^ We found no association signal with specific ischemic stroke subtypes, possibly related to limited power. *DYRK1A* maps to the Down syndrome (DS) critical genetic region and is thought to contribute to the manifestations of DS. Recent work has drawn attention to an increased risk of stroke in DS.^20^ Although this might relate to other factors, our findings in conjunction with the above experimental data suggest a link between *DYRK1* and stroke.

As a limitation, we were not able to perform meta‐analyses for LAS, CES, and SVS with UKB because information on etiological subtypes of ischemic stroke is not yet available in UKB. Also, the gain in power compared to MEGASTROKE was limited because of the relatively small number of stroke cases (around 5,000) in UKB, although numbers will increase with further follow‐up.^13^ Nonetheless, we found 3 novel loci, providing further insights into relevant stroke pathways and mechanisms. Integration of additional datasets will be key to better understanding the genetic basis of stroke.

## Author Contributions

R.M., K.R., J.C.H., C.L.M.S., S.D., and M.D. were involved in the conception and the design of the study. All authors acquired and analyzed the data. R.M. and M.D. drafted the manuscript. R.M., M.D., K.R., C.L.M.S., M.S., and S.D. made critical revisions to the manuscript.

## Potential Conflicts of Interest

Nothing to report.
